# Skp2 Expression Is Inhibited by Arsenic Trioxide through the Upregulation of miRNA-330-5p in Pancreatic Cancer Cells

**DOI:** 10.1016/j.omto.2019.01.006

**Published:** 2019-02-05

**Authors:** Jiankun Gao, Gu Wang, Jingrong Wu, Yu Zuo, Jing Zhang, Xintian Jin

**Affiliations:** 1 Department of Basic Medical Science, Sichuan College of Traditional Chinese Medicine, Mianyang, Sichuan 621000, China; 2 Department of Thoracic Oncosurgery, Jilin Province Cancer Hospital, Changchun, Jilin 130012, China

**Keywords:** arsenic trioxide, Skp2, miRNA-330-5p, pancreatic cancer, cell growth

## Abstract

Arsenic trioxide (ATO) has been found to exert its anti-cancer activity in various human malignancies. In our previous report, we have shown that ATO inhibited cell growth and invasion via downregulation of Skp2 in pancreatic cancer (PC) cells. It has been extensively demonstrated that microRNAs (miRNAs) play a pivotal role in tumorigenesis. ATO might induce PC cell apoptosis and regulate Skp2 downregulation through the regulation of miRNAs. One study has demonstrated that miR-330-5p exerts a tumor-suppressive function in PC cell lines. Here, we investigated the role of miRNA-330-5p in ATO-mediated anti-tumor activity and explored whether ATO could regulate miR-330-5p in PC cells. We found that ATO treatment upregulated the expression of miR-330-5p. Moreover, miR-330-5p inhibitor rescued the ATO-mediated tumor-suppressive function. The combination of miR-330-5p mimic with ATO reduced cell growth, motility, and invasion, and enhanced apoptosis to a greater degree in PC cells. This study suggests that the combination of miR-330-5p mimic with ATO may be a potential therapeutic strategy for the treatment of PC.

## Introduction

Pancreatic cancer (PC) is one of the lethal malignant tumors with high morbidity and mortality. In the United States, approximately 55,440 new cases of PC and 44,330 deaths were expected in 2018.^1^ The morbidity and mortality of PC in China have also gradually increased in recent years.^2^ Current therapies for PC are involved in surgery and chemotherapy. For most advanced PC patients, who missed the surgery time, chemotherapy is the preferred approach to offer symptomatic relief and extend the median survival modestly. The standard chemotherapy was gemcitabine alone or in combination with other chemotherapeutic agents;^3^ however, the efficacy is still limited. Improved therapeutic strategies are urgently needed.

Arsenic trioxide (ATO) is a chemotherapeutic agent with clinical effects, which have been widely used in hematopoietic malignancy, especially for acute promyelocytic leukemia treatment.^4^ ATO has been observed to have promising activities in various solid tumors.^5^ The F-box protein Skp2 (S-phase kinase associated protein 2) plays a critical role in the development and progression of PC.^6^ Skp2 promotes ubiquitin-mediated proteolysis of its substrates, including p21, p27, p57, E-cadherin, and Foxo1 (forkhead box O1), leading to its oncogenic role in tumorigenesis.^7^, ^8^, ^9^ We previously found that ATO inhibited PC cell growth and migration through downregulation of Skp2 expression.^10^ However, the molecular mechanism by which ATO inhibits Skp2 remains unclear. ATO might regulate Skp2 expression through the regulation of microRNA (miRNA) expression.

The miRNAs are single-stranded, short, noncoding RNAs 20–24 nt in length that post-transcriptionally regulate the expression of their target mRNAs.^11^ Increasing evidence indicates that miRNAs play critical roles in a wide range of biological processes, such as proliferation, differentiation, and apoptosis, which link them with numerous human diseases, including cancer.^12^ One study has demonstrated that miR-330-5p is a tumor suppressor in PC cell lines.^13^ Although several target genes are known for miR-330-5p,^14^, ^15^, ^16^ the exact molecular mechanisms of its involvement in cancer are not fully elucidated. We therefore decided to further investigate the role of miRNA-330-5p in ATO-mediated anti-tumor activity and explored whether there was the synergistic effect between miR-330-5p and ATO on cell growth, migration, invasion, and apoptosis in PC cells.

## Results

### ATO Upregulates miR-330-5p Expression in PC Cells

**Figure 1:**
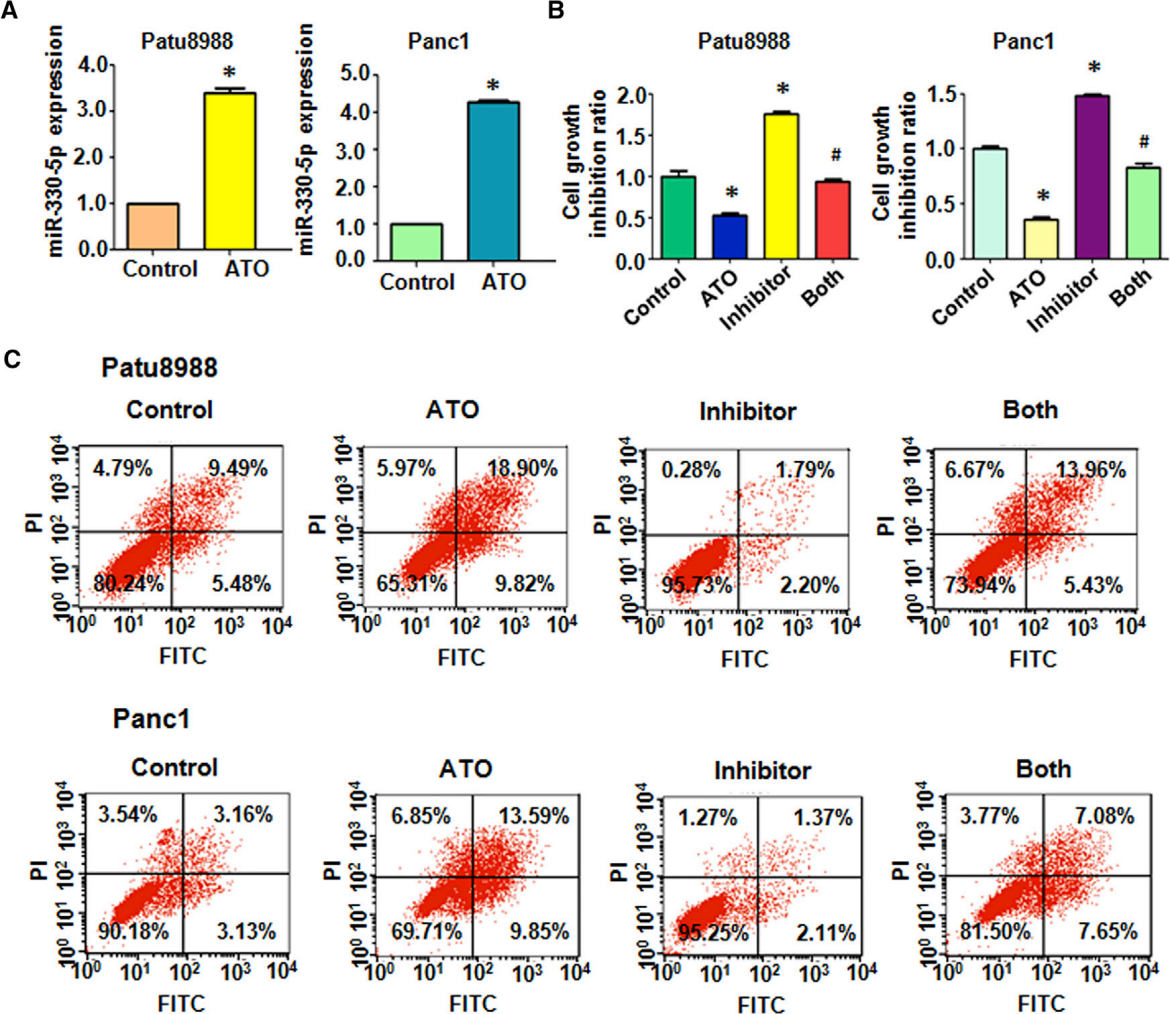
Effect of ATO and miR-330-5p Inhibitor on Cell Growth and Apoptosis (A) Real-time RT-PCR was performed to measure miR-330-5p expression in PC cells treated with 3 μmol/L ATO for 72 h. (B) MTT assay was conducted to detect cell proliferation in PC cells after 3 μmol/L ATO or miR-330-5p inhibitor or the combination for 72 h. (C) Apoptotic cell death was measured using Annexin V-FITC and propidium iodide method in PC cells after 3 μmol/L ATO or miR-330-5p inhibitor or the combination for 72 h.

To verify whether ATO could regulate miR-330-5p expression in PC cells, we treated the cells with 3 μmol/L ATO in Patu8988 and Panc-1 cells. Then we performed real-time RT-PCR analysis. The data showed that miR-330-5p expression level was increased obviously when cells were treated with ATO in both PC cell lines (Figure 1A).

### ATO and miR-330-5p Govern the Viability of PC Cells

We have previously reported that 3 μmol/L ATO inhibited cell growth in Patu8988 and Panc-1 cells.^10^ In order to further investigate the influence of miR-330-5p on PC cell growth, we evaluated the cell viability in PC cells transfected with miR-330-5p inhibitor or in combination with ATO for 48 h by MTT assay. The results showed that the miR-330-5p inhibitor effectively promoted cell growth (Figure 1B). Moreover, our results showed that ATO inhibited PC cell growth, whereas miR-330-5p inhibitor promoted PC cell growth. The growth-inhibiting effect was stronger in the ATO alone group than in the ATO plus miR-330-5p inhibitor group (Figure 1B). These results demonstrated that ATO inhibited cell growth partly via upregulation of miR-330-5p.

### ATO and miR-330-5p Induce Apoptosis in PC Cells

To determine whether miR-330-5p induces apoptosis in PC cells, we performed the cell apoptosis assay using Annexin V and PI (propidium iodide) staining. We used flow cytometry to investigate the level of apoptosis and analyzed the combined effect of ATO and the miR-330-5p inhibitor. We found that miR-330-5p inhibitor led to a decrease in the rate of apoptosis in PC cells. The percentage of late apoptotic cells decreased from 9.49% in the control to 1.79% and from 3.16% in control cells to 1.37% in miR330-5p inhibitor transfected Patu8988 and Panc-1 cells, respectively (Figure 1C). The cells treated with ATO alone showed a significant increase in the late apoptotic rate (18.90% of Patu8988 cells, 13.59% of Panc-1 cells), and the cells treated with ATO after transfection with the miR-330-5p inhibitor showed a greater degree of decrease in late apoptosis (13.96% of Patu8988 cells, 7.08% of Panc-1 cells) compared with the ATO treatment alone group (Figure 1C).

### ATO and miR-330-5p Inhibit PC Cell Migration

**Figure 2:**
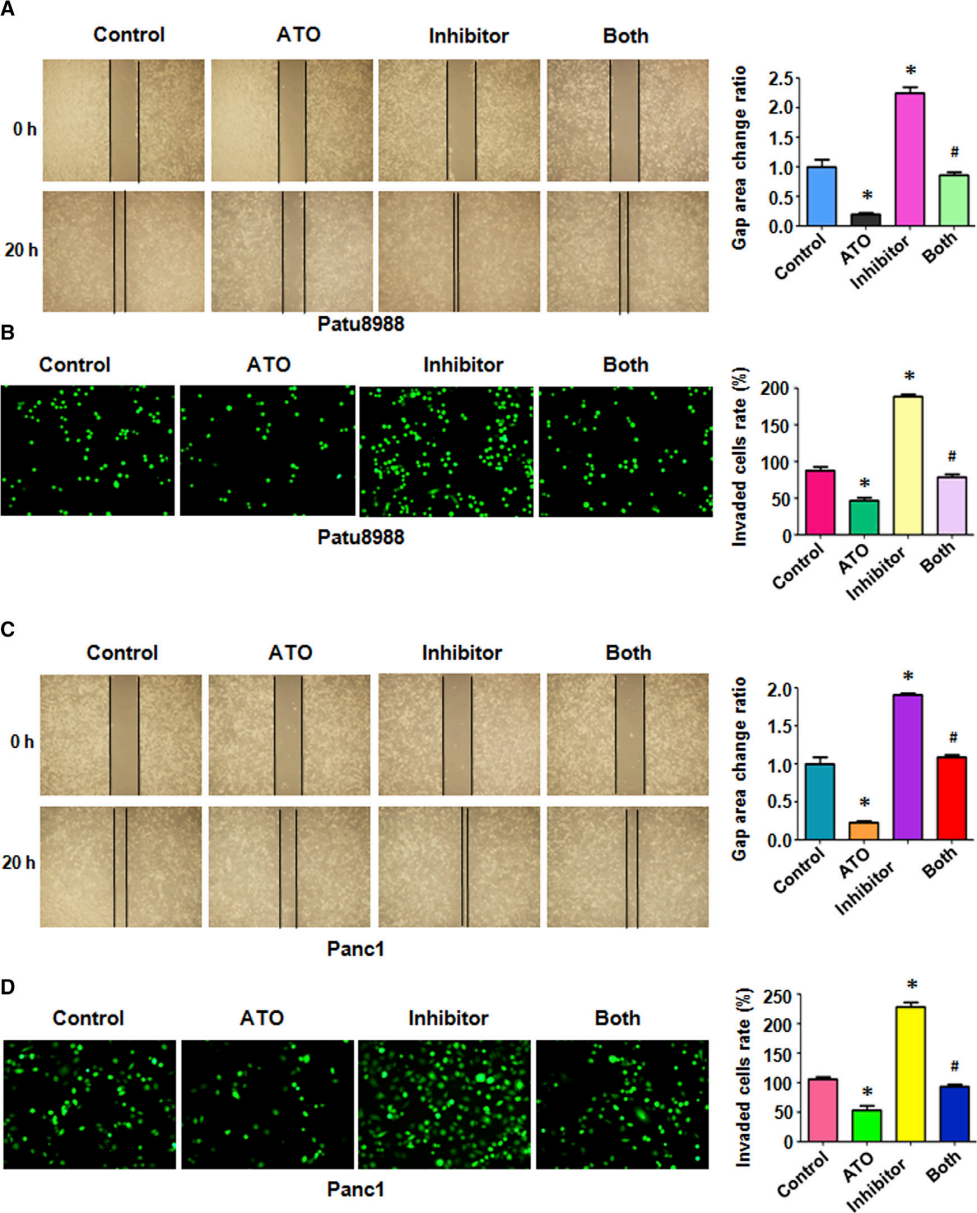
Effect of ATO and miR-330-5p Inhibitor on Cell Migration and Invasion (A) Left: cell migration was detected using wound-healing assay in Patu8988 cells after 3 μmol/L ATO or miR-330-5p inhibitor or the combination for 20 h. Right: quantitative results were illustrated for left image. (B) Left: cell invasion was measured using Transwell inserts with Matrigel in Patu8988 cancer cells after 3 μmol/L ATO or miR-330-5p inhibitor or the combination for 20 h. Right: quantitative results were illustrated for left image. (C) Left: cell migration was detected using wound-healing assay in Panc-1 cells after 3 μmol/L ATO or miR-330-5p inhibitor or the combination for 20 h. Right: quantitative results were illustrated for left image. (D) Left: cell invasion was measured using Transwell inserts with Matrigel in Panc-1 cells after 3 μmol/L ATO or miR-330-5p inhibitor or the combination for 20 h. Right: quantitative results were illustrated for left image. *p < 0.05 versus control; ^#^p < 0.05 versus ATO treatment or miR-330-5p inhibitor transfection.

In order to examine whether ATO and miR-330-5p could prevent migratory activity in Panc-1 and Patu8988 cells, we conducted wound-healing assays in cells treated with or without ATO and analyzed the combined effect of ATO and the miR-330-5p inhibitor. As shown in Figures 2A and 2C, the wound closure rate was significantly increased in the miR-330-5p inhibitor transfected cells compared with the control cells. The cells treated with ATO alone showed a decrease in the wound closure rate, and the cells treated with ATO after transfection with the miR-330-5p inhibitor showed a remarkable increase compared with the ATO alone group (Figures 2A and 2C). Next, we investigated whether ATO and miR-330-5p could inhibit cell invasion in PC cells.

### ATO and miR-330-5p Inhibit PC Cell Invasion

To further confirm the function of ATO and miR330-5p in cell motility, we measured the cell invasion capacities in transfected or non-transfected PC cells with ATO treatment using Matrigel invasion chamber assay. We found that miR330-5p inhibitor promoted the cell invasion in both Patu8988 and Panc-1 cells (Figures 2B and 2D), and the cells treated with ATO after transfection with the miR-330-5p inhibitor showed an increase in the invasion rate compared with the ATO alone group (Figures 2B and 2D). These data indicated that ATO and miR-330-5p could inhibit cell invasion in PC cells.

### miR-330-5p Inhibitor Increased Skp2 Protein Expression

**Figure 3:**
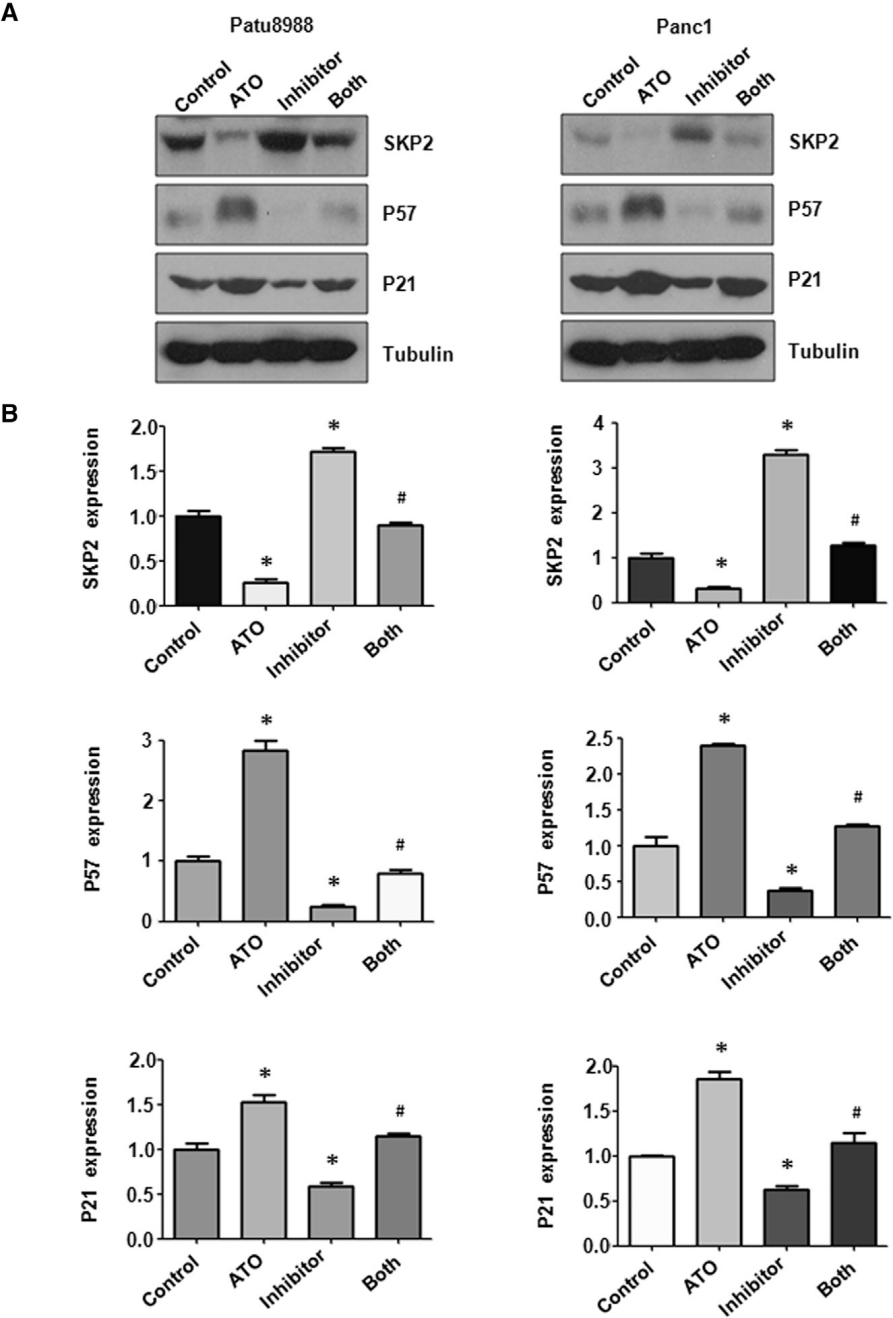
Effect of ATO and miR-330-5p Inhibitor on the Expression of Skp2 (A) The expression of Skp2, p57, and p21 was measured by western blotting analysis in PC cells after ATO treatment or miR-330-5p inhibitor or the combination. (B) Quantitative results are illustrated for (A). *p < 0.05 versus control; ^#^p < 0.05 versus ATO treatment or miR-330-5p inhibitor transfection.

Our initial study indicated that ATO regulates the expression of Skp2 in PC cells.^10^ To determine whether miR-330-5p also regulates the expression of Skp2, we investigated the effects of the miR-330-5p inhibitor on Skp2 expression at protein level. The amount of protein in the transfected PC cells was analyzed using western blotting analysis. The results showed that the miR-330-5p inhibitor increased the expression of Skp2 (Figures 3A and 3B), Moreover, the inhibition of Skp2 expression by ATO treatment was rescued by miR-330-5p inhibitor treatment in PC cells (Figures 3A and 3B).

It has been reported that tumor suppressor p21 is a substrate of Skp2.^17^ Additionally, Skp2 has been found to attenuate p57 function in human cancers.^18^ In line with these findings, our results revealed that ATO treatment enhanced accumulation of tumor suppressor p57 and p21 in PC cells (Figures 3A and 3B). Furthermore, our results showed that the miR-330-5p inhibitor decreased the expression of p57 and p21. Moreover, the upregulation of p57 and p21 expression by ATO treatment was partly blocked by the miR-330-5p inhibitor transfection group (Figures 3A and 3B).

### miR-330-5p Mimic Inhibits Cell Growth and Induces Apoptosis

**Figure 4:**
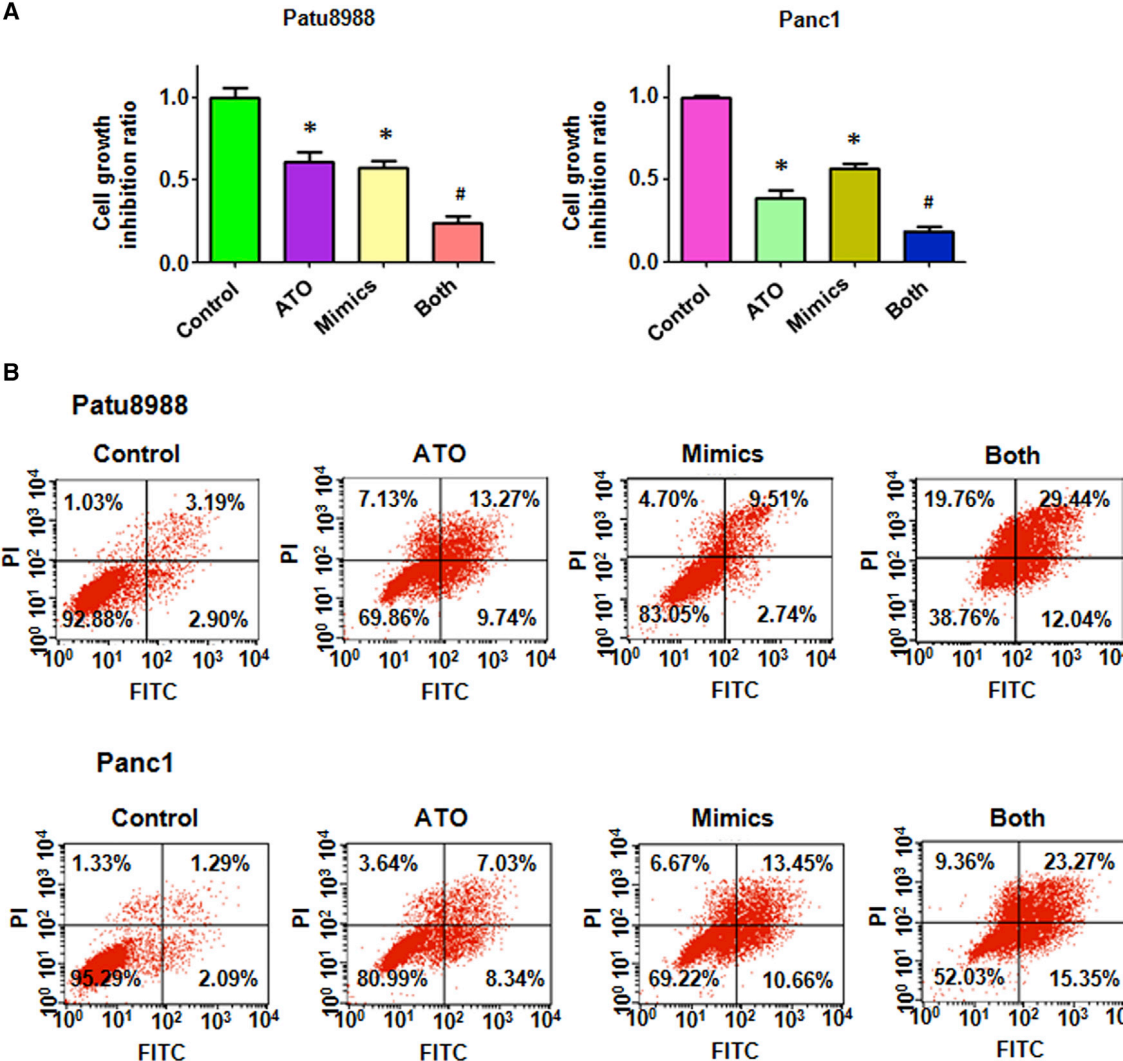
Effect of ATO and miR-330-5p Mimic on Cell Growth and Apoptosis (A) MTT assay was conducted to detect cell proliferation in PC cells after 3 μmol/L ATO or miR-330-5p mimic or the combination for 72 h. *p < 0.05 versus control; ^#^p < 0.05 versus ATO treatment or miR-330-5p mimic transfection. (B) Apoptotic cell death was measured using the Annexin V-FITC and propidium iodide method in PC cells after 3 μmol/L ATO or miR-330-5p mimic or the combination for 72 h.

Our study further revealed that miR-330-5p inhibited cell growth in PC cells (Figure 4A). Moreover, cell growth inhibition was stronger in the ATO in combination with miR-330-5p mimic group than that in the ATO alone group (Figure 4A). In addition, miR-330-5p mimic led to increases in the rate of apoptosis in PC cells. The percentage of late apoptotic cells increased from 3.19% in the control to 9.51% and from 1.29% in control cells to 13.45% in miR-330-5p mimic transfected Patu8988 and Panc-1 cells (Figure 4B). The cells treated with ATO after transfection with the miR-330-5p mimic showed an increased late apoptosis compared with the ATO alone group (Figure 4B), suggesting that ATO and miR-330-5p mimic induce apoptosis additively in PC cells.

### miR-330-5p Mimic Inhibits PC Cell Migration and Invasion

**Figure 5:**
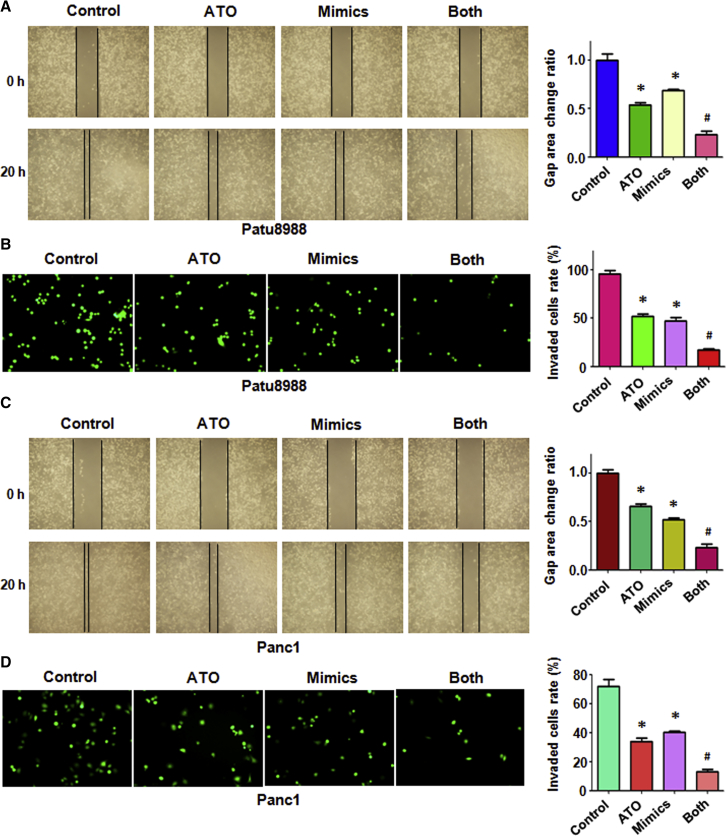
Effect of ATO and miR-330-5p Mimic on Cell Migration and Invasion (A) Left: cell migration was detected using wound-healing assay in Patu8988 cancer cells after 3 μmol/L ATO or miR-330-5p mimic or the combination for 20 h. Right: quantitative results were illustrated for the left image. (B) Left: cell invasion was measured using Transwell inserts with Matrigel in Patu8988 cancer cells after 3 μmol/L ATO or miR-330-5p mimic or the combination for 20 h. Right: quantitative results were illustrated for the left image. (C) Left: cell migration was detected using wound-healing assay in Panc-1 cancer cells after 3 μmol/L ATO or miR-330-5p mimic or the combination for 20 h. Right: quantitative results were illustrated for the left image. (D) Left: cell invasion was measured using Transwell inserts with Matrigel in Panc-1 cancer cells after 3 μmol/L ATO or miR-330-5p mimic or the combination for 20 h. Right: quantitative results were illustrated for the left image. *p < 0.05 versus control; ^#^p < 0.05 versus ATO treatment or miR-330-5p mimic transfection.

Our wound-healing assay results showed that miR-330-5p mimic inhibited cell migration in PC cells (Figures 5A and 5C). The cells treated with ATO after transfection with the miR-330-5p mimic showed a decrease compared with the ATO alone group (Figures 5A and 5C). These data indicated that ATO and miR-330-5p could inhibit migration additively of PC cells. Our invasion assay data showed that ATO and miR-330-5p mimic significantly retarded the cells’ invasion (Figures 5B and 5D). The cells treated with ATO after transfection with the miR-330-5p mimic showed a decrease compared with the ATO alone group (Figures 5B and 5D).

### miR-330-5p Mimic Inhibits Skp2 Protein Expression

**Figure 6:**
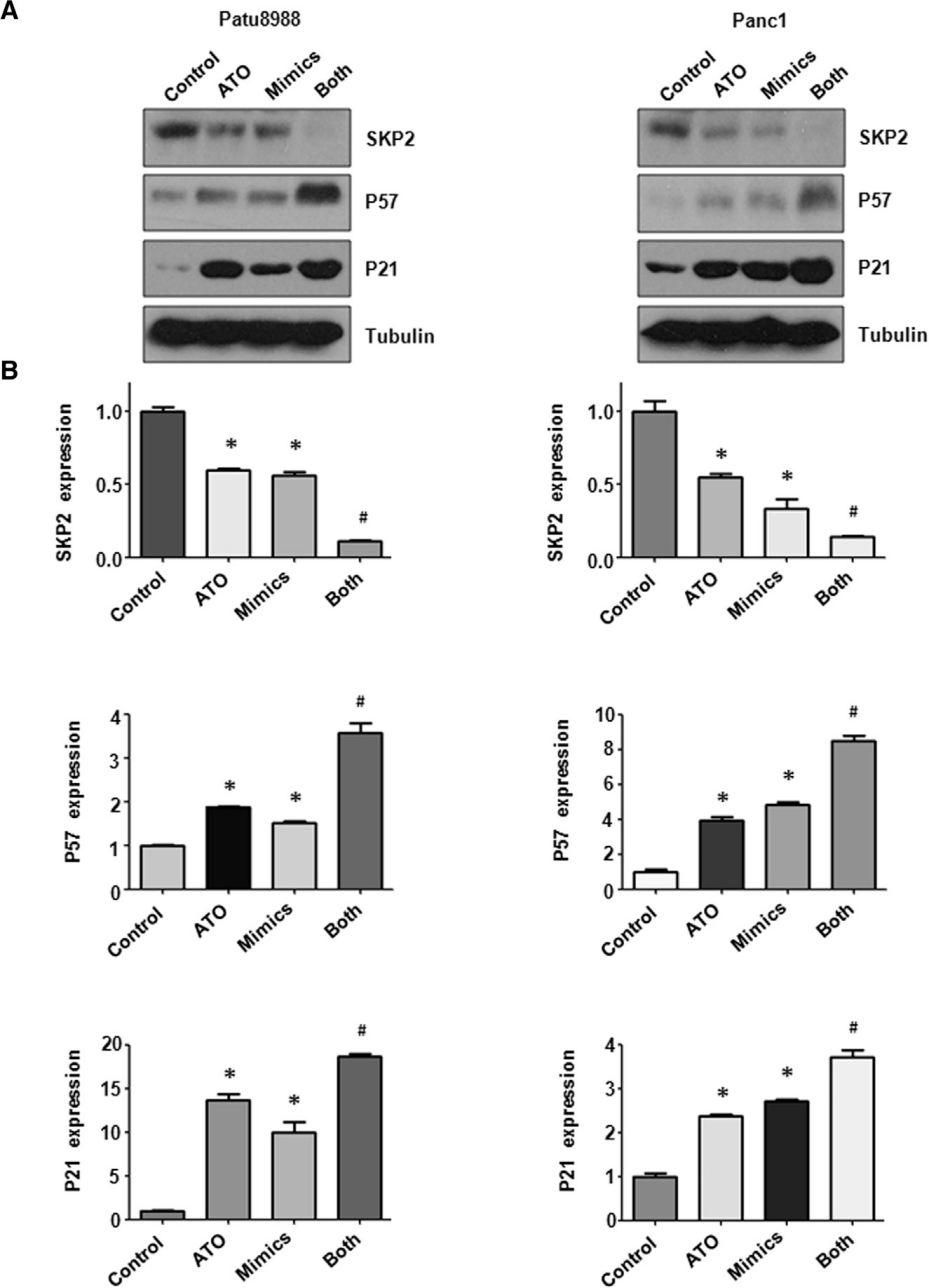
Effect of ATO and miR-330-5p Mimic on the Expression of Skp2 (A) The expression of Skp2, p57, and p21 was measured by western blotting analysis in PC cells after ATO treatment or miR-330-5p mimic or the combination. (B) Quantitative results are illustrated for (A). *p < 0.05 versus control; ^#^p < 0.05 versus ATO treatment or miR-330-5p mimic transfection.

Our western blotting results showed that the miR-330-5p mimic inhibited the expression of Skp2 in PC cells (Figures 6A and 6B). Consistently, miR-330-5p mimic increased the expression of p57 and p21 (Figures 6A and 6B). Moreover, the level of Skp2 was lower in cells treated with ATO and miR-330-5p mimic compared with ATO alone or mimic alone (Figures 6A and 6B). In line with this, p57 and p21 expressions were higher in the ATO plus miR-330-5p mimic group (Figures 6A and 6B).

## Discussion

A number of studies have demonstrated that ATO inhibited cell growth and induced apoptosis in PC cells.^19^, ^20^, ^21^ Furthermore, Han et al.^22^ found that ATO inhibited viability of PC stem cells *in vitro* and *in vivo* via binding to SHH (sonic hedgehog)-Gli. In our study, we also found that ATO inhibited the proliferation of PC cells. Our data support earlier studies showing that ATO can inhibit Skp2 expression in PC cells.^10^ Several studies have highlighted the critical role of Skp2 in human cancer progression including pancreatic carcinogenicity.^23^, ^24^ Chan et al.^25^ reported that Skp2-SCF E3 ligase regulated Akt ubiquitination, glycolysis, Herceptin sensitivity, and tumorigenesis. Consistently, an Skp2 inhibitor, SZL-P1-41, has been shown to restrict cancer stem cell traits and cancer progression.^26^ Similarly, a novel selenonucleoside, LJ-2618, targeted Skp2 degradation and suppressed tumor growth in paclitaxel-resistant prostate cancer.^27^ Thus, Skp2 might be a promising therapeutic molecular target in human cancers including PC.^28^, ^29^, ^30^

Many experiments have revealed that the expression of Skp2 is regulated by miRNAs. For example, miR-30 family postponed metastasis via targeting Skp2 in lung cancer cells *in vitro* and *in vivo*.^31^ In addition, miR-3163 as a mediator of Skp2 regulation inhibited cell growth in lung cancer.^32^ Tréhoux et al.^13^ found that miR-330-5p is a tumor-suppressive miRNAs in PC cells. Several studies have also identified the important role of miR-330-5p in human cancers. For example, miR-330-5p was characterized as a putative modulator of neoadjuvant chemoradiotherapy sensitivity in esophageal adenocarcinoma.^33^ In addition, miR-330-5p was found as a negative regulator of T cell immunoglobulin and mucin domain-3 (TIM-3) in acute myeloma leukemia (AML) cells.^34^ Moreover, miR-330-5p regulated tyrosinase and PDIA3 expression, leading to inhibition of cell proliferation and invasion in cutaneous malignant melanoma.^35^ Furthermore, miR-330-5p negatively regulated integrin α5 expression in colorectal cancer and glioblastoma.^16^, ^36^ Kong et al.^15^ found that miR-330-5p inhibited NOB1 and repressed cell growth in non-small cell lung cancer. Recently, miR-330-5p was found to target Sprouty2 and to promote cancer progression through the mitogen-activated protein kinase-extracellular signal regulated kinase (MAPK-ERK) signaling pathway in hepatocellular carcinoma.^37^ We used a specific miRNA-330-5p inhibitor and a miR-330-5p mimic to study the potential mechanisms underlying the effects of ATO in PC cells. Our data revealed that ATO significantly upregulated miRNA-330-5p expression and downregulated Skp2. Transfection with the miR-330-5p inhibitor upregulated Skp2, whereas miR-330-5p mimic downregulated Skp2. Importantly, downregulation of miR-330-5p by its inhibitor partly rescued ATO-mediated anti-tumor activity. The data indicated that ATO exerts its tumor-suppressive function in part via upregulation of miR-330-5p and subsequent inhibition of Skp2 in PC cells. Further investigation is required to determine whether ATO exhibits anti-cancer activity in mouse models via upregulation of miR-330-5p. It is interesting whether PC patients have low expression of miR-330-5p.

In summary, upregulation of miR-330-5p could be a promising way to treat patients with PC. Furthermore, the combination of miR-330-5p mimic and ATO could be a potential therapeutic strategy for PC patients. It must be noted that using ATO and miR-330-5p mimic in clinic for PC patients has a long way to identify the treatment benefit.

## Materials and Methods

### Cell Culture and Experiment Reagents

Human PC cell lines Patu8988 and Panc-1 were obtained from ATCC and were cultured in DMEM supplemented with 10% (v/v) FBS, 100 μg/mL streptomycin, and 100 U/mL penicillin and in standard cell culture conditions containing 5% CO_2_ at 37°C in a humidified atmosphere. Antibodies against Skp2, P57, P21, Tubulin, and the secondary antibodies were obtained from Santa Cruz Biotechnology (Santa Cruz, CA, USA). ATO and all other chemicals were bought from Sigma (St. Louis, MO, USA). ATO was dissolved in 1 mM NaOH to make 1 mM stock solution and was added directly to the media at different concentrations.

### Oligonucleotide Design and Synthesis

The miR-330-5p mimic and miR-330-5p inhibitor oligonucleotides were designed using sequences that were complementary to mature miR-330-5p. All the oligodeoxynucleotides were chemically synthesized (GenePharma, Shanghai, China).

### RNA Extraction and Real-Time PCR

The levels of miR-330-5p mRNA were measured in the Pac-1 and Patu8988 cell lines at 48 h after the 3 μmol/L ATO treatments by real-time RT-PCR assay as described previously.^38^

### ATO Treatment and miRNA Transfection

Panc-1 and Patu8988 cells in the exponential growth phase were seeded in six-well plates. The cells were transfected with miR-330-5p inhibitor and miR-330-5p mimic. All transfections were performed according to the manufacturer’s instructions as described previously.^39^ After transfection with the miR-330-5p inhibitor or the miR-330-5p mimic, either ATO or medium alone was added, and the transfected cells were cultured for an additional 48 h.

### MTT Assay

The effect of ATO, miR330-5p inhibitor or miR-330-5p mimic transfection, or the combination on PC cell growth was analyzed using MTT assay. In brief, the cells were seeded in a 96-well culture plate and incubated for 48 h in the absence or presence of 3 μmol/L ATO. At the end of the treatment period, 10 μL of Reagent MTT (5 mg/mL in PBS) was added to each well. After 2-h incubation, 100 μL of DMSO was added to each well and further incubated for 10 min in the dark. The color intensity was measured by SpectraMax M5 microplate fluorometer (Molecular Devices, USA) at 490 nm.

### Annexin V-FITC Method for Apoptosis Analysis

Annexin V-FITC (fluorescein isothiocyanate) apoptosis detection kit (Biouniqure, China) was used to measure the apoptotic cells. The miR-330-5p inhibitor or miR-330-5p mimic transfected PC cells were incubated in the presence or absence of 3 μmol/L ATO for 48 h. Cells were collected by centrifugation and then were resuspended in 500 μL of binding buffer. After that, 5 μL of Annexin V-FITC and 5 μL of propidium iodide were added. All of the samples were kept in the dark for 15 min at room temperature. Finally, the stained cells were analyzed using a flow cytometer (BD Biosciences, USA).

### Wound-Healing Assay

In brief, miR330-5p inhibitor or miR-330-5p mimic transfected PC cell migrations were detected using a wound-healing assay. The wound was generated in the cells with 90%–95% confluence by scratching the surface of the plates with a sterile pipette tip. The cells were then incubated in the absence or presence of 3 μmol/L ATO for 20 h and then photographed with an inverted phase-contrast microscopy (Olympus, Japan).

### Transwell Invasion Assay

To measure the invasive capacity of PC cells, we performed miR-330-5p inhibitor or miR-330-5p mimic transfected PC cells using Transwell Filter (8-μm pore size; Corning, USA) with Matrigel (BD Biosciences, USA). In brief, cells in serum-free media were transferred in each upper chamber in the presence or absence of 3 μmol/L ATO. Then 0.5 mL of culture medium with 10% FBS was added into each bottom chamber in the presence or absence of 3 μmol/L ATO. After incubation for 20 h, the cells in the upper chamber were removed (the upper surfaces of the Transwell chambers were scraped with cotton swabs), and the invaded cells were fixed and stained using the Wright’s-Giemsa stain set (Jiancheng Scientific, China). The stained cells were photographed and counted under a light microscope in six randomly selected fields.

### Protein Extraction and Western Blotting

For protein extraction, cells were harvested and lysed with cell lysis buffer (Cell Signaling, USA). The protein concentrations were measured using the bicinchoninic acid (BCA) protein assay kit. Proteins were fractionated using SDS-PAGE, and the gels were transferred onto nitrocellulose membrane. The membranes were blocked with 4% nonfat dried milk or BSA in 1× PBS containing 0.1% Tween 20 and then incubated overnight at 4°C with appropriate primary antibodies. The membranes were washed three times with PBS-T and subsequently incubated with the secondary antibodies for 1 h at room temperature. The protein bands were detected using the enhanced chemiluminescence detection system.

### Data Analysis

Data are represented as mean ± SD for the absolute values or percentage of controls as indicated in the vertical axis legend of figures. The statistical significance of differential findings between experimental groups and control groups was statistically evaluated using one-way ANOVA followed by Tukey’s post hoc test by GraphPad StatMate software (GraphPad Software, USA). The p value <0.05 was considered statistically significant.

## Author Contributions

J.G. and X.J. performed the experiments, analyzed the data, and wrote the manuscript. G.W., J.W., and Y.Z. performed the experiments and analyzed the data. J.Z. analyzed the data.
